# Serum Albumin and Prealbumin Predict the Poor Outcome of Traumatic Brain Injury

**DOI:** 10.1371/journal.pone.0093167

**Published:** 2014-03-26

**Authors:** Du Chen, Long Bao, Shi-qi Lu, Feng Xu

**Affiliations:** Department of Emergency Medicine, the First Affiliated Hospital of Soochow University, Suzhou, China; University of Leicester, United Kingdom

## Abstract

**Background:**

Serum albumin and prealbumin are both negative acute-phase reactants, and usually at low levels in stress. We aim to determine their predictive values for poor outcome of traumatic brain injury (TBI).

**Methods:**

A total of 326 patients of TBI were enrolled and followed-up by telephone 6 months after discharge. They were divided into a favorable group (GOS: 3 to 5) and an unfavorable group (GOS: 1 to 2). Serum albumin and prealbumin were measured from vein blood within 24 h after admission.

**Results:**

Ninety one (27.9%) patients were with poor outcome (GOS: 1 to 2). The unfavorable group had lower albumin and prealbumin (*P*<0.001). Albumin and prealbumin were both positively correlated with GCS (r = 0.489, *P*<0.001; r = 0.222, *P*<0.001, respectively) and GOS (r = 0.518, *P*<0.001; r = 0.314, *P*<0.001, respectively). After adjustment for confounding factors, the odds ratios of albumin and prealbumin were 0.866, 95% CI: 0.829 to 0.904 and 0.990, 95% CI: 0.985 to 0.995, respectively. In subgroup of GCS≤8 (*n* = 101), the crude and adjusted odds ratios of serum albumin were both statistically significant (*P* = 0.027, *P* = 0.033, respectively), while prealbumin were not (*P* = 0.553, *P* = 0.576, respectively). The AUC of albumin for predicting poor outcome was 0.762, 95% CI: 0.712 to 0.807, which was significantly higher than that of prealbumin (0.664, 95% CI: 0.610 to 0.715). In analyses of all patients and subgroup of GCS≤8, the AUCs of serum albumin were both significantly higher than those of prealbumin (*P* = 0.001, *P* = 0.045, respectively).

**Conclusions:**

Both serum albumin and prealbumin could predict the poor outcome of TBI, but the former is much better, especially, in patients with severe TBI.

## Introduction

Traumatic brain injury (TBI) is a major global health problem. Country-based estimates of incidence range from 108 to 332 new cases admitted to hospital per 100 000 population per year. On average, 39% of patients with severe TBI die from their injury, and 60% have an unfavorable outcome on the Glasgow Outcome Scale (GOS) [1]. According to the World Health Organization (WHO) in high-income countries TBI is the leading cause of death under the age of 40. Based on the prognosis of the WHO, in two decades, head injury will be the third most frequent cause of death in the world [2]. The socioeconomic consequences of TBI have ignited widespread research aimed at decreasing the burden of the disease. A major field to be explored is the establishment and construction of reliable prognostic tools.

Albumin and prealbumin are both indicators of protein nutrition status. Like albumin, prealbumin is also a negative acute-phase reactant reducing in stress, and both of them could be used as predictors for prognosis of some diseases. To our knowledge, there have been few studies to compare their predictive values for poor outcome of TBI.

The aim of this study is to investigate the predictive values of serum albumin and prealbumin for the poor outcome of TBI through a retrospective case-control study, and make a comparison between them.

## Materials and Methods

### Patients

A total of 326 patients with TBI [male: 229 (70.3%), age: 49.1±24.9 years] were enrolled from the emergency department or intensive care unit of the First Affiliated Hospital of Soochow University, a tertiary teaching hospital, between November 2010 and October 2012. Inclusion criteria were: TBI within 24 hours; and Glasgow Coma Scale (GCS) 3 to 14. Patients <18 years old, pregnant women, and patients with autoimmune disease were excluded. Patients' characteristics, such as age, sex, GCS, and clinical factors that may affect the levels of serum albumin and prealbumin were collected. The levels of serum albumin and prealbumin were measured from fasting venous blood within 24 h after admission. Analyses were performed by Olympus AU2700 automatic biochemical Analyzer.

For prognosis analysis, patients were followed-up by telephone 6 months after discharge. Each follow-up examination or interview was recorded. According to these data, patients were divided into 5 groups based on GOS [3]: 1, death; 2, vegetative state – unable to interact with environment; 3, severe disability – unable to live independently; 4, moderate disability – capable of independent living but unable to return work or school; 5, complete recovery – able to participate in work or school. Patients were then classified into a favorable group with high GOS (3 to 5) and an unfavorable group with low GOS (1 to 2).

### Ethics Statement

The study was carried out according to the Helsinki Declaration and approved by the ethical committee of the First Affiliated Hospital of Soochow University. The need for informed consent was waived, because the data used in this study had already been collected for clinical purposes. Furthermore, the present study did not interfere with the treatment of patients and the database was organized in a way that makes the identification of an individual patient impossible.

### Statistical analyses

Categorical data were presented as frequency and percentage (%), and measurement data with normal distribution and non-normal distribution were presented as mean ±SD or median (*P*_25_, *P*_75_), respectively. Comparisons of continuous variables were performed using the Mann-Whitney *U* test or unpaired *t* test, while the chi-square test was applied for categorical variables,and the Bonferroni method was used for multiple comparisons. The independent predictors of TBI were determined by univariate and multivariate logistic regression; odds ratios (OR) and 95% CI were calculated. The correlations between predictors and GCS, GOS were assessed by Spearman correlation, while Pearson correlation was used for serum albumin and prealbumin. The Receiver Operating Characteristic (ROC) curves were plotted for predictors, and the area under the curve (AUC) was used to evaluate the predictive value. The AUCs were compared by DeLong method [4]. Statistical analyses were performed by Stata version 12 (StataCorp, Texas, USA). *P*<0.05 was considered as statistical significance (two-tailed).

## Results

### Analyses of baseline data

The outcomes of all patients: death, 81 (24.85%); vegetative state, 10 (3.07%); severe disability, 16 (4.91%); moderate disability, 84 (25.77%); complete recovery, 135 (41.41%). Of the 326 patients with TBI, ninety one paients were of death or vegetative state (unfavorable group). As table 1 illustrated, there were significant differences in sex, age, GCS, pupil reactivity, injury severity score, days in hospital, and the levels of serum albumin and prealbumin between unfavorable and favorable groups. Especially, the unfavorable group had significantly lower levels of albumin and prealbumin, higher percentage of male, older age, poorer pupil reactivity, and lower GCS. There were no significant differences in clinical factors (aspiration or resuscitation, multiple contusions and skin burnings) between the two groups (*P*>0.05).

**Table 1:** Demographic and clinical characteristics in unfavorable and favorable groups.

Variable	Unfavorable (*n* = 91)	Favorable (*n* = 235)	*P* value
	(GOS: 1–2)	(GOS: 3–5)	
Sex (*n*/%)			0.032^a^
Male	56 (61.5)	173 (73.6)	
Female	35 (38.5)	62 (26.4)	
Age (year)	55.3±20.4	46.0±16.6	<0.001^b^
GCS	5 (3, 8)	13 (11, 14)	<0.001^c^
Pupil reactivity (*n*/%)			<0.001^a^
Both reacting	29	216	
One reacting	22	17	
Both non-reacting	40	2	
ISS	26 (25, 33)	13 (9, 20)	<0.001^c^
Days in hospital (day)	10 (5, 22)	10 (7, 14)	0.700^c^
Clinical factors (*n*/%)			
Aspiration or resuscitation	8 (3.3)	13 (3.3)	0.282 a
Multiple contusions	79 (86.8)	185 (82.1)	0.319 a
Skin burnings	3 (3.3)	4 (1.7)	0.373 a
Albumin (g/L)	31.9 (26.2, 37.7)	34.7 (38.7, 42.9)	<0.001^c^
Prealbumin (mg/L)	136.9 (104.2, 181.1)	168.4 (132.0, 209.0)	<0.001^c^

GCS, Glasgow Coma Scale; ISS, Injury Severity Score; GOS, Glasgow Outcome Scale;

a chi-square test;

b *t* test;

c Mann-Whitney *U* test.

### Correlation analyses

**Figure 1:**
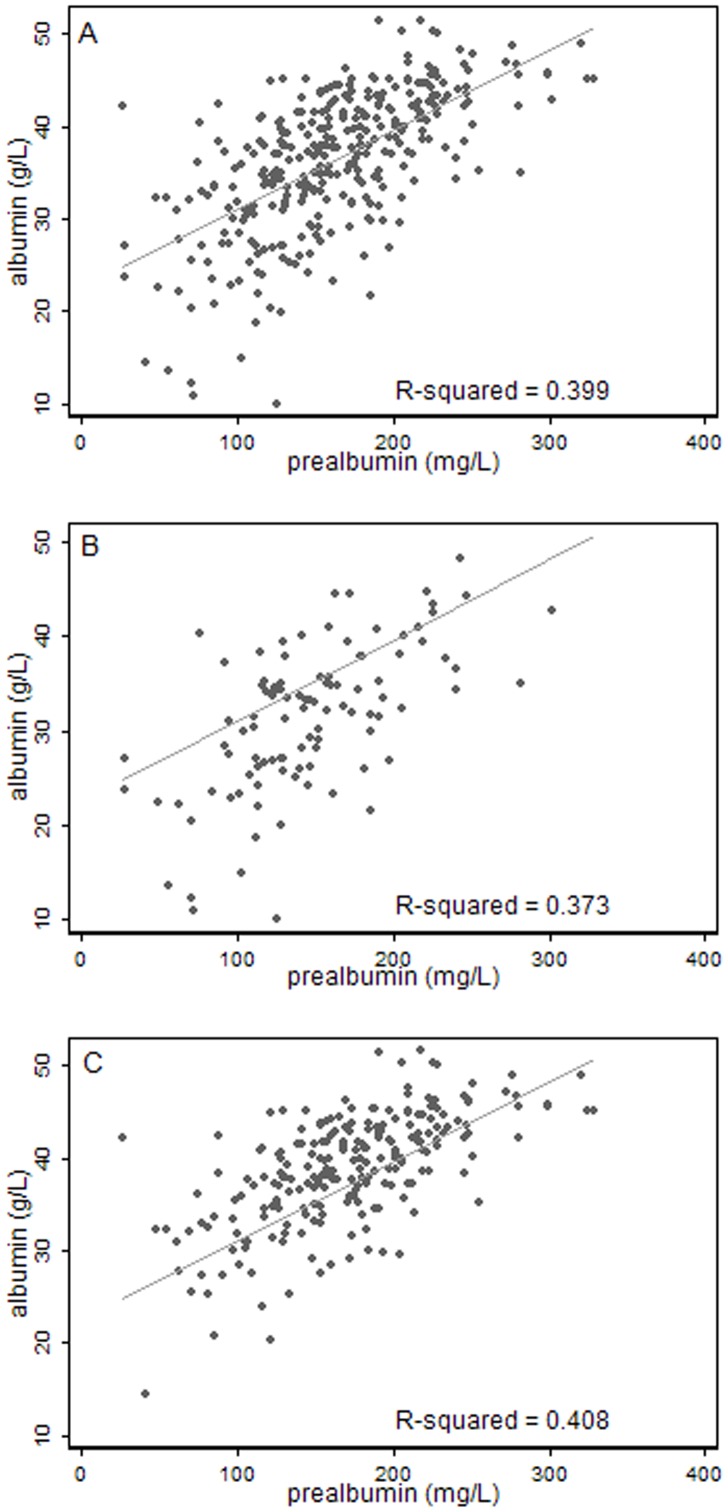
Scatter plot of serum albumin and prealbumin. A: all patients; B: subgroup of GCS≤8; C: subgroup of GCS>8. Subgroup analyses indicated that the correlation was relatively stable. The level of serum albumin had a moderate correlation with that of prealbumin (r = 0.650, *P*<0.001) in all patients.

**Figure 2:**
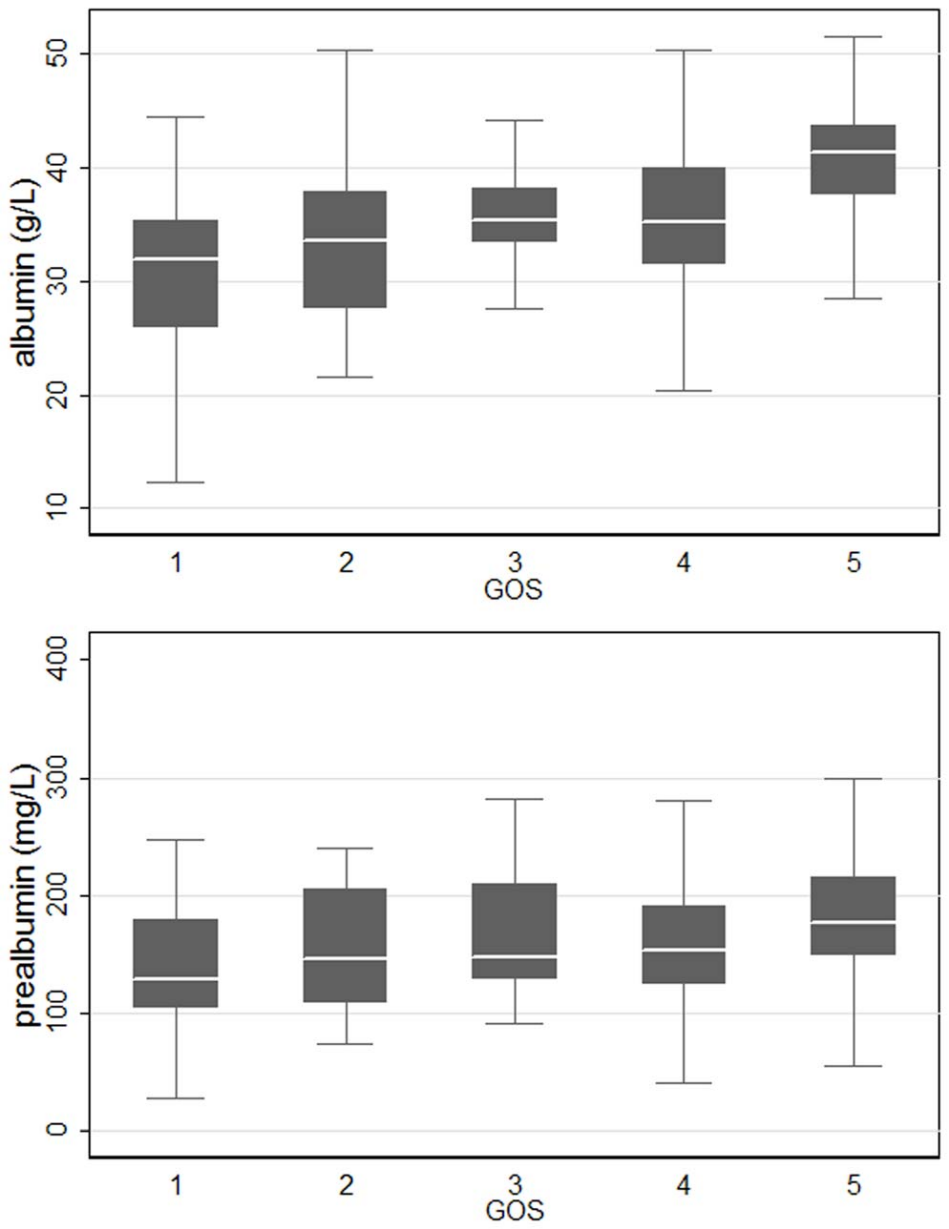
Box plots of serum albumin and prealbumin in GOS subgroups. Serum albumin and prealbumin were both rising with GOS, and the former was more obvious (r _albumin_ = 0.518, *P*<0.001; r _prealbumin_ = 0.314, *P*<0.001).

The scatter plot of serum albumin and prealbumin displayed a moderate positively correlation (r = 0.650, *P*<0.001) in all patients, and subgroup analyses indicated the correlation was relatively stable (Figure 1). Both of them were positively correlated with GCS (r _albumin_ = 0.489, *P*<0.001; r _prealbumin_ = 0.222, *P*<0.001) and GOS (r _albumin_ = 0.518, *P*<0.001; r _prealbumin_ = 0.314, *P*<0.001). As Figure 2 illustrated, serum albumin had a more obvious correlation with GOS.

### Subgroup analyses of unfavorable outcome

**Figure 3:**
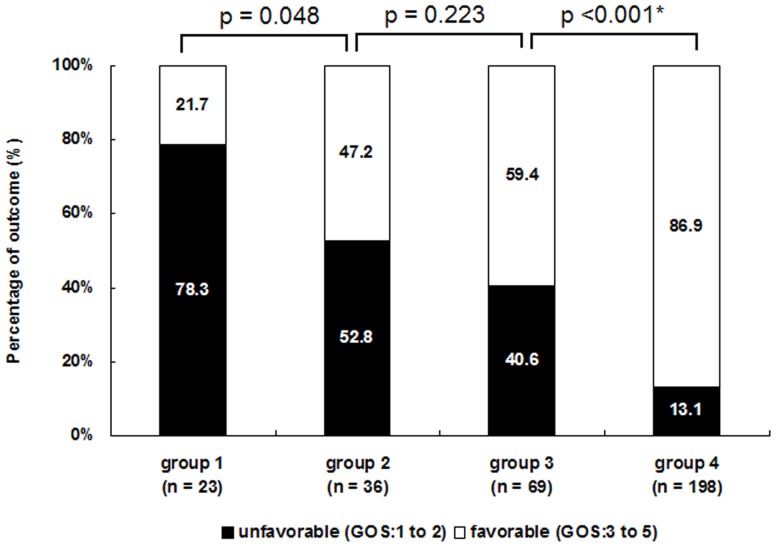
Percentage plot displaying percentages of unfavorable outcome in four subgroups with different levels of serum albumin. (group 1: <25 g/L; group 2: 25 to 30 g/L; group 3: 30 to 35 g/L; group 4: ≥35 g/L) From group 1 to group 4, the percentages of unfavorable outcome (GOS: 1 to 2) displayed a downward trend (Cochran-Armitage trend test, *P*<0.001), but only the difference between group 3 and group 4 was statistically significant. (α after Bonferroni correction  = 0.008. ^*^ Statistically significant)

According to the serum albumin level, all patients were stratified into four subgroups (group 1: <25 g/L;group 2: 25 to 30 g/L; group 3: 30 to 35 g/L; group 4: ≥35 g/L). In each subgroup, percentages of the unfavorable outcome were 78.3%, 52.8%, 40.6%, and 13.1%, respectively; and the plot demonstrated a downward trend (Cochran-Armitage trend test, *P*<0.001) (Figure 3). Chi-square test indicated there were statistically significances in percentages of the unfavorable outcome in the four groups (*P*<0.001). Furthermore, we performed pair-wise comparisons with the alpha correction (α after Bonferroni correction  = 0.008), and a significant difference was found between group 3 and group 4 (*P*<0.001).

### Logistic regression analyses

In the analysis of all patients, both univariate and multivariate logistic regressions determined serum albumin and prealbumin as independent predictors for poor outcome of TBI (Table 2), and the adjusted odds ratios were 0.866, 95% CI: 0.829 to 0.904 and 0.990, 95% CI: 0.985 to 0.995, respectively. Furthermore, subgroup analyses were performed. In subgroup of GCS≤8 (*n* = 101), the crude and adjusted odds ratios of serum albumin were both statistically significant (*P* = 0.027 and *P* = 0.033, respectively), while those of prealbumin were both not (*P* = 0.553 and *P* = 0.576, respectively). In subgroup of GCS>8 (*n* = 225), only crude odds ratios of serum albumin and prealbumin were statistically significant (*P* = 0.032 and *P*<0.001, respectively), while the adjusted odds ratios of them were not (*P* = 0.596 and *P* = 0.082, respectively).

**Table 2:** Logistic regression analyses for predictors.

Predictor	Univariate logistic regression		Multivariate logistic regression	
	Crude OR (95% CI)	*P* value	Adjusted a OR (95% CI)	*P* value
Albumin (g/L)				
All patients (*n* = 326)	0.870 (0.835 to 0.906)	<0.001	0.866 (0.829 to 0.904)	<0.001
GCS≤8 (*n* = 101)	0.931 (0.874 to 0.992)	0.027	0.927 (0.865 to 0.994)	0.033
GCS>8 (*n* = 225)	0.925 (0.861 to 0.993)	0.032	0.975 (0.888 to 1.070)	0.596
Prealbumin (mg/L)				
All patients (*n* = 326)	0.989 (0.984 to 0.994)	<0.001	0.990 (0.985 to 0.995)	<0.001
GCS≤8 (*n* = 101)	0.997 (0.989 to 1.006)	0.553	0.997 (0.988 to 1.006)	0.576
GCS>8 (*n* = 225)	0.981 (0.971 to 0.991)	<0.001	0.989 (0.977 to 1.001)	0.082

OR, odds ratio; CI, confident interval; GCS, Glasgow Coma Scale;

a Adjusted for age, sex and clinical factors using logistic regression model.

### Analyses of ROC curves

**Figure 4:**
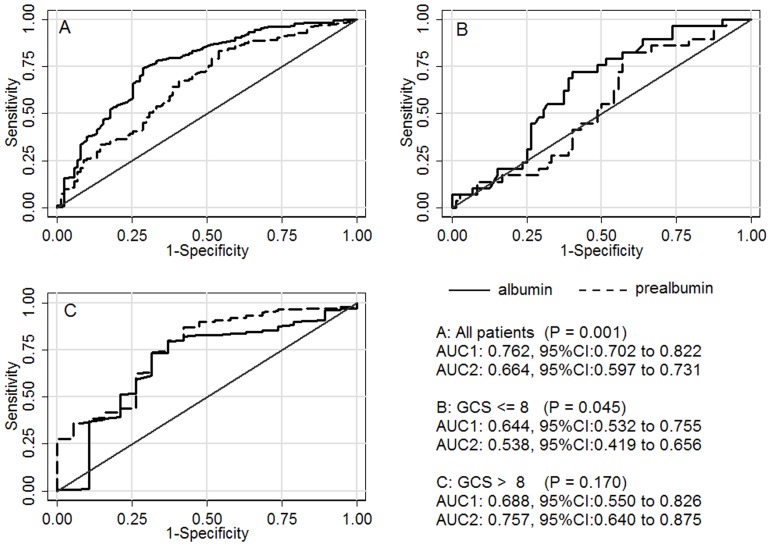
ROC curve analyses of different groups of TBI. AUC1 and AUC2 are on behalf of the areas under the ROC curves of serum albumin and prealbumin, respectively. In all patients and subgroup of GCS≤8, the AUCs of serum albumin were significantly higher than those of prealbumin (*P* = 0.001 and *P* = 0.045, respectively), while in the subgroup of GCS>8, no significant differences of AUCs were observed (*P* = 0.170). Furthermore, the AUCs of serum albumin were statistically significant in both subgroups (*P*<0.05), while prealbumin showed no predictive value in the subgroup of GCS≤8 (AUC = 0.538, 95%CI: 0.419 to 0.656, *P*>0.05).

The AUC of serum albumin for predicting unfavorable outcome (GOS: 1 to 2) of TBI was 0.762, 95% CI: 0.712 to 0.807, which was significantly higher than that of prealbumin (0.664, 95% CI: 0.610 to 0.715). As Figure 4 displayed, in all patients and subgroup of GCS≤8, the AUCs of serum albumin were significantly higher than those of prealbumin (*P* = 0.001 and *P* = 0.045, respectively), while in the subgroup of GCS>8, no significant differences of AUCs were found (*P* = 0.170). In addition, the AUCs of serum albumin were statistically significant in both subgroups (*P*<0.05), while prealbumin showed no predictive value in the subgroup of GCS≤8 (AUC = 0.538, 95% CI: 0.419 to 0.656, *P*>0.05).

## Discussion

### Findings from results

In the logistic regression analyses of all patients, serum albumin and prealbumin were both statistically significant after adjustment for age, sex and clinical factors, but in subgroup analyses, different performances of them were observed. In the subgroup of GCS≤8, serum albumin was still determined as an independent predictor, while prealbumin was not. In the subgroup of GCS>8, in univariate logistic regression, serum albumin and prealbumin both displayed associations with unfavorable outcome, but in multivariate analyses, both of them did not have statistical significance (*P*>0.05) after adjustment for age and sex. In ROC curve analyses, the AUCs of serum albumin were statistically significant in both subgroups (*P*<0.05), while prealbumin showed no predictive value in the subgroup of GCS≤8. These phenomena may be related to their different roles in TBI and the different pathophysiological processes of mild and severe subgroups.

### Comparison of physiological function between albumin and prealbumin

Albumin and prealbumin, products from the liver, are both indicators of nutrition status, and could be used as predictors of prognosis. They have some differences in physiological functions [5]. a) Albumin has a relatively long half-life of approximately 20 days and a very large serum pool. b) Albumin is a negative acute-phase reactant. This means that albumin concentrations rise slowly during nutritional therapy and in patients recovering from stress. c) Liver function should be considered while evaluating albumin levels. d) Zinc's main transport vehicle in the blood is albumin. Prealbumin is another protein status indicator. It has a much shorter half-life and smaller serum pool compared to albumin. The half-life of prealbumin is approximately two days, making prealbumin a more timely and sensitive indicator of protein status. Prealbumin is a tryptophan-rich protein, and like albumin, it is synthesized in the hepatocytes of the liver. Like albumin, prealbumin is also a negative acute-phase reactant [5]. Low levels could result from either inadequate nutrition or inflammatory stress.

### Role of albumin and prealbumin in TBI

In patients with heart failure, cancer, and cerebral infarction, prealbumin has been shown to be a good predictor of prognosis [6], [7], [8]. It has been reported that serum prealbumin also has predictive value for TBI [9]. In our study, the ability of prealbumin to predict the prognosis of TBI seems not as good as that of albumin, especially in patients with severe TBI (GOS≤8).

There are different mechanisms explaining the decline of serum albumin in patients with TBI. Firstly, albumin consumption is increased under stress state. Secondly, hemorrhage caused albumin lost. Thirdly, inadequate intake and the suppressed liver function would reduce albumin synthesis. In addition, albumin extravasation due to increased vascular permeability and blood-brain barrier dysfunction. The blood brain barrier (BBB) is composed of vascular endothelium, basal lamina, pericytes and astrocyte foot processes anchored by tight junctions [10]. The BBB prevents fluid, macromolecules, and small molecules from exiting the microvasculature and entering the brain parenchyma. When the integrity of the BBB is compromised in patients with TBI, infiltration is one of the ways that result in the albumin reduction. Rossi and colleagues performed an experiment implicating albumin, acting through p38 mitogen activated protein kinase, in a novel mechanism by which activation of myosin light chain kinase following TBI may lead to compromise of the BBB [11]. This finding explains the mechanism of reduced albumin which leads to the poor prognosis of TBI from the aspect of the blood-brain barrier integrity. In addition, albumin also has many other important physiological functions. Lower level of albumin is bound to weaken the patient's resistance, resulting in slow wound healing and fragile resistance to secondary infection. Eventually, the risk of poor prognosis is significantly increasing. Previous studies reported that low level of serum albumin seems to be an independent predictor for poor outcome of TBI [12], [13], and results of our study give this a further confirmation.

### Debate about albumin administration and our viewpoint

It has been proven that low levels of albumin are associated with a poor outcome, but for the patients of TBI, whether albumin level should be elevated and what is the best level are still controversial. This is just like glucose control in TBI. Lots of studies determine that hyperglycemia is an independent risk factor for TBI [14], [15], [16], [17], [18], [19], but it does not mean that to reduce the glucose level will improve prognosis [20], [21], [22], [23]. Similarly, albumin supplement for patients of TBI is another problem researchers should investigate. A clinical trial [24] reported that in critically ill patients with TBI, fluid resuscitation with albumin was associated with higher mortality rates than with saline. Actually, the use of albumin for resuscitation in patients with severe TBI could increase intracranial pressure during the first week, and this is the most likely mechanism of increased mortality in these patients [25]. On the contrary, there are also a few of studies holding different viewpoints [26], [27]. For example, Rodling and colleagues reported that a protocol including albumin administration in combination with a neutral to a slightly negative fluid balance was associated with low mortality in patients with severe TBI [27]. Recently, Yang and colleagues performed a retrospective cohort study, and concluded that the most favorable level of albumin for uncomplicated severe TBI is 29–31 g/L [26]. We preformed a retrospective case-control study, and the results demonstrated, in patients of TBI with serum albumin ≥35 g/L, the proportion of unfavorable outcome reduced significantly. Findings from animal experiment may explain the mechanism partly. In the experiment, it was observed that high-concentration albumin therapy instituted 15 min after trauma significantly improves the neurological score and reduces histological damage [28]. To sum up, based on related studies and our results, low level of serum albumin is a risk factor of TBI, and the percentage of unfavorable outcome decreases with the increase of serum albumin. Although this issue is still controversial, we believe that it would be better to maintain a moderately high level of serum albumin for patients of TBI.

### Limitations

There are a few of limitations in our study. Firstly, it is a retrospective study from only one medical center. Secondly, the sample size is not big, and may be with selection bias. Thirdly, this case-control study could not directly answer the questions that if albumin resuscitation is reasonable and what is the optimal level of serum albumin to pursue in clinical practice. Prospective clinical trials are needed to answer these questions.

### Conclusions

In conclusion, both serum albumin and prealbumin may be used as predictors for unfavorable outcome of TBI, but the performances are significantly different, and former is much better. Especially, in patients of severe TBI (GCS≤8), serum albumin is determined as an independent predictor, while prealbumin seems to be with no predictive value.

## References

[1] Rosenfeld JV, Maas AI, Bragge P, Morganti-Kossmann MC, Manley GT, et al (2012) Early management of severe traumatic brain injury. Lancet 380: 1088–1098. 22998718 10.1016/S0140-6736(12)60864-2

[2] Czeiter E, Mondello S, Kovacs N, Sandor J, Gabrielli A, et al (2012) Brain injury biomarkers may improve the predictive power of the IMPACT outcome calculator. J Neurotrauma 29: 1770–1778. 22435839 10.1089/neu.2011.2127 PMC3409455

[3] Lingsma HF, Roozenbeek B, Steyerberg EW, Murray GD, Maas AI (2010) Early prognosis in traumatic brain injury: from prophecies to predictions. Lancet Neurol 9: 543–554. 20398861 10.1016/S1474-4422(10)70065-X

[4] DeLong ER, DeLong DM, Clarke-Pearson DL (1988) Comparing the areas under two or more correlated receiver operating characteristic curves: a nonparametric approach. Biometrics 44: 837–845. 3203132

[5] Collins N (2001) The difference between albumin and prealbumin. Adv Skin Wound Care 14: 235–236. 11905970 10.1097/00129334-200109000-00009

[6] Cabassi A, Champlain JD, Maggiore U, Parenti E, Coghi P, et al.. (2013) Prealbumin improves death risk prediction of BNP-added Seattle Heart Failure Model: Results from a pilot study in elderly chronic heart failure patients. Int J Cardiol. 10.1016/j.ijcard.2013.04.039 23623341

[7] Kawai H, Ota H (2012) Low perioperative serum prealbumin predicts early recurrence after curative pulmonary resection for non-small-cell lung cancer. World J Surg 36: 2853–2857. 22948197 10.1007/s00268-012-1766-y

[8] Gao C, Zhang B, Zhang W, Pu S, Yin J, et al (2011) Serum prealbumin (transthyretin) predict good outcome in young patients with cerebral infarction. Clin Exp Med 11: 49–54. 20535523 10.1007/s10238-010-0103-8

[9] Rocca B, Bidet PF, Courtinat C, Lecoq SH, Chevalier A, et al (1987) [Lack of prognostic value of the determination of 3 serum proteins during the acute phase of brain injury]. Ann Fr Anesth Reanim 6: 476–481. 2450492 10.1016/s0750-7658(87)80091-6

[10] Yurchenco PD, Schittny JC (1990) Molecular architecture of basement membranes. FASEB J 4: 1577–1590. 2180767 10.1096/fasebj.4.6.2180767

[11] Rossi JL, Ralay RH, Patel F, Chrzaszcz M, Venkatesan C, et al (2011) Albumin causes increased myosin light chain kinase expression in astrocytes via p38 mitogen-activated protein kinase. J Neurosci Res 89: 852–861. 21360574 10.1002/jnr.22600 PMC3079319

[12] Bernard F, Al-Tamimi YZ, Chatfield D, Lynch AG, Matta BF, et al (2008) Serum albumin level as a predictor of outcome in traumatic brain injury: potential for treatment. J Trauma 64: 872–875. 18404050 10.1097/TA.0b013e31803428cc

[13] Nelson DW, Rudehill A, MacCallum RM, Holst A, Wanecek M, et al (2012) Multivariate outcome prediction in traumatic brain injury with focus on laboratory values. J Neurotrauma 29: 2613–2624. 22994879 10.1089/neu.2012.2468

[14] Smith RL, Lin JC, Adelson PD, Kochanek PM, Fink EL, et al (2012) Relationship between hyperglycemia and outcome in children with severe traumatic brain injury. Pediatr Crit Care Med 13: 85–91. 21499170 10.1097/PCC.0b013e3182192c30 PMC3677026

[15] Seyed SS, Bidabadi E, Seyed SS, Mashouf M, Salamat F, et al (2012) Association of persistent hyperglycemia with outcome of severe traumatic brain injury in pediatric population. Childs Nerv Syst 28: 1773–1777. 22526446 10.1007/s00381-012-1753-5

[16] Matsushima K, Peng M, Velasco C, Schaefer E, Diaz-Arrastia R, et al (2012) Glucose variability negatively impacts long-term functional outcome in patients with traumatic brain injury. J Crit Care 27: 125–131. 22033047 10.1016/j.jcrc.2011.08.012

[17] Melo JR, Di Rocco F, Blanot S, Laurent-Vannier A, Reis RC, et al (2010) Acute hyperglycemia is a reliable outcome predictor in children with severe traumatic brain injury. Acta Neurochir (Wien) 152: 1559–1565. 20461419 10.1007/s00701-010-0680-z

[18] Jiang JY, Gao GY, Li WP, Yu MK, Zhu C (2002) Early indicators of prognosis in 846 cases of severe traumatic brain injury. J Neurotrauma 19: 869–874. 12184856 10.1089/08977150260190456

[19] Rovlias A, Kotsou S (2000) The influence of hyperglycemia on neurological outcome in patients with severe head injury. Neurosurgery 46: : 335–342, 342–343. 10.1097/00006123-200002000-00015 10690722

[20] Kramer AH, Roberts DJ, Zygun DA (2012) Optimal glycemic control in neurocritical care patients: a systematic review and meta-analysis. Crit Care 16: R203. 23082798 10.1186/cc11812 PMC3682305

[21] Vespa P, McArthur DL, Stein N, Huang SC, Shao W, et al (2012) Tight glycemic control increases metabolic distress in traumatic brain injury: a randomized controlled within-subjects trial. Crit Care Med 40: 1923–1929. 22610193 10.1097/CCM.0b013e31824e0fcc

[22] Shutter L (2012) Glucose control in traumatic brain injury: extra sweetness required. Crit Care Med 40: 1995–1996. 22610222 10.1097/CCM.0b013e3182514c15

[23] Sharma D, Vavilala MS (2012) Perioperative management of adult traumatic brain injury. Anesthesiol Clin 30: 333–346. 22901613 10.1016/j.anclin.2012.04.003 PMC3424485

[24] Myburgh J, Cooper DJ, Finfer S, Bellomo R, Norton R, et al (2007) Saline or albumin for fluid resuscitation in patients with traumatic brain injury. N Engl J Med 357: 874–884. 17761591 10.1056/NEJMoa067514

[25] Cooper DJ, Myburgh J, Heritier S, Finfer S, Bellomo R, et al (2013) Albumin resuscitation for traumatic brain injury: is intracranial hypertension the cause of increased mortality? J Neurotrauma 30: 512–518. 23194432 10.1089/neu.2012.2573 PMC3636581

[26] Yang TJ, Fei MM, Ye W, Pan AJ, Liu B (2013) [Effect of albumin and hemoglobin level on prognosis of patients with uncomplicated severe traumatic brain injury: a retrospective cohort study]. Zhonghua Wei Zhong Bing Ji Jiu Yi Xue 25: 301–305. 23663583 10.3760/cma.j.issn.2095-4352.2013.05.016

[27] Rodling WM, Olivecrona M, Nystrom F, Koskinen LO, Naredi S (2009) Fluid therapy and the use of albumin in the treatment of severe traumatic brain injury. Acta Anaesthesiol Scand 53: 18–25. 18945246 10.1111/j.1399-6576.2008.01798.x

[28] Belayev L, Alonso OF, Huh PW, Zhao W, Busto R, et al (1999) Posttreatment with high-dose albumin reduces histopathological damage and improves neurological deficit following fluid percussion brain injury in rats. J Neurotrauma 16: 445–453. 10391362 10.1089/neu.1999.16.445

